# Bully Victimization: Selection and Influence Within Adolescent Friendship Networks and Cliques

**DOI:** 10.1007/s10964-015-0343-8

**Published:** 2015-09-01

**Authors:** Gerine M. A. Lodder, Ron H. J. Scholte, Antonius H. N. Cillessen, Matteo Giletta

**Affiliations:** Behavioural Science Institute, Radboud University, P.O. Box 9104, 6500 HE Nijmegen, The Netherlands; Praktikon, P.O. Box 9104, 6500 HE Nijmegen, The Netherlands; Department of Developmental Psychology, Tilburg University, P.O. Box 90153, 5000 LE Tilburg, The Netherlands

**Keywords:** Bully victimization, Adolescence, Friendship networks, Cliques, Selection, Influence

## Abstract

Adolescents tend to form friendships with similar peers and, in turn, their friends further influence adolescents’ behaviors and attitudes. Emerging work has shown that these selection and influence processes also might extend to bully victimization. However, no prior work has examined selection and influence effects involved in bully victimization within cliques, despite theoretical account emphasizing the importance of cliques in this regard. This study examined selection and influence processes in adolescence regarding bully victimization both at the level of the entire friendship network and the level of cliques. We used a two-wave design (5-month interval). Participants were 543 adolescents (50.1 % male, Mage = 15.8) in secondary education. Stochastic actor-based models indicated that at the level of the larger friendship network, adolescents tended to select friends with similar levels of bully victimization as they themselves. In addition, adolescent friends influenced each other in terms of bully victimization over time. Actor Parter Interdependence models showed that similarities in bully victimization between clique members were not due to selection of clique members. For boys, average clique bully victimization predicted individual bully victimization over time (influence), but not vice versa. No influence was found for girls, indicating that different mechanisms may underlie friend influence on bully victimization for girls and boys. The differences in results at the level of the larger friendship network versus the clique emphasize the importance of taking the type of friendship ties into account in research on selection and influence processes involved in bully victimization.

## Introduction

Bully victimization refers to the process by which an adolescent is repeatedly and over time exposed to intentional negative actions by their peers, and can include physical, verbal or relational aggression (Hamburger et al. 2011; Olweus 1996). Bully victimization can be distinguished from fighting or teasing by an imbalance in power between bully and victim (Olweus 1996). Bully victimization is distinguished from other forms of victimization because of a power difference between the perpetrator and the victim (Salmivalli and Peets 2009). Bully victimization is prevalent across countries worldwide, with an average of about 11 % of children reporting being bully victimized (Currie and Organization 2000). Bully victimization is a very powerful stressor in adolescence and can have long-lasting physical and psychological consequences (Arseneault et al. 2010). Recent work has shown that bullying and bully victimization should be understood as a group phenomenon (Salmivalli 2010). Besides the adolescents who bully and the victims of bullying, other peers are also involved in bullying by, for example, defending the victim or reinforcing the adolescents who bully (Salmivalli et al. 1996). Research also has begun to recognize that adolescents who belong to the same peer group might resemble each other in terms of how much they bully or are bully victimized by others (Espelage et al. 2007; Faris and Felmlee 2014; Huitsing et al. 2014; Sentse et al. 2013; Sijtsema et al. 2013).

Friends tend to be similar in a wide variety of behaviors (Brechwald and Prinstein 2011). These similarities can be due to selection or influence (Veenstra and Dijkstra 2011). Selection is the processes by which individuals choose friends who resemble themselves on certain characteristics. Influence is the processes that increase similarity between individuals once they have established a relationship (e.g., friendship) (Veenstra et al. 2013). Earlier research on children and adolescents examined selection and influence effects in the larger friendship network, taking into account all friendship ties within a school (Sentse et al. 2013; Sijtsema et al. 2013). One of these studies differentiated between relational victimization (e.g., being excluded) and overt victimization (e.g., being hit) (Sijtsema et al. 2013). Results indicated that adolescents who were relationally victimized tended to select friends who were similarly relationally victimized. Influence effects occurred for both relational and overt victimization. Other research found evidence for selection effects on overt victimization, and influence effects on relational victimization (Sentse et al. 2013). Thus, at the level of the larger friendship network, both selection and influence effects on bully victimization seem to occur, implying that not only individual characteristics of adolescents who are bully victimized are important for the development of bully victimization, but group processes play an important role (Salmivalli 2010). Indeed, many prevention and intervention programs aimed at reducing bully victimization work with peers, for instance, by creating a support group around children who are bully victimized, and show promising results (Ttofi and Farrington 2009).

Earlier research suggested that not all peers equally influence adolescents, and in some cases influence seems to be stronger in close friendships than more distant friendships in the larger friendship network (Giletta et al. 2012). If indeed peers who are closer to the adolescent have larger influence on their bully victimization, these peers may be important to target in bullying interventions. It is, therefore, important to examine whether selection and influence occur at different levels of the peer network, for instance by examining these effects at the level of the larger friendship network and at the level of closer friendships. Adolescents have multiple dyadic friendships that differ in their level of closeness (e.g., best friendships and close friendships). These friendships are interconnected in more complex friendship structures, such as groups, ultimately forming what can be referred to as the larger adolescent friendship network (Scholte and Van Aken 2006). Within these large networks of friendships, cliques can be defined as exclusive and relatively tight groups of friends with whom adolescents spend most of their time (Brown 2004; Brown and Klute 2006; Henrich et al. 2000). Compared to other peers that are more distantly connected to adolescents, clique members are considered to be among the most important sources of influence on adolescent development (Adler and Adler 1998; Brown and Klute 2006; Thompson et al. 2001; Witvliet et al. 2010b). A growing involvement in cliques occurs during the adolescent years (Thompson et al. 2001). Adolescents experience more affect, intimacy and self-disclosure with close friends, and during adolescence friendship groups such as cliques become more important for adjustment than in childhood (Giordano 2003). Because of the large role clique members play in adolescent development, researchers emphasized the importance of cliques when it comes to selection and influence processes (e.g., Conway et al. 2011; Ennett and Bauman 1994; Espelage et al. 2007; Paxton et al. 1999). Within the larger friendship network, cliques have their own social norms, and may thus have a different influence on adolescents compared to other peers in the larger friendship network (Urberg et al. 1995). Earlier research indicated that dyadic best friends may be more important compared to other peers in the larger friendship network (Giletta et al. 2012). However, because dyadic friendships usually do not occur in isolation, but are embedded within cliques, examining the added effect of all clique members is crucial (Bagwell et al. 2000; Espelage et al. 2007). Earlier research has not yet examined selection and influence processes involved in bully victimization at the level of the clique. But, earlier research showed that selection and influence processes at the level of the clique play a role for the perpetration of bullying (Espelage et al. 2007; Witvliet et al. 2010a). In addition, there is evidence that clique members resemble each other in their levels of bully victimization, but it is unknown whether this similarity is related to selection or peer influence processes (Salmivalli et al. 1997). Thus, despite the possible importance of cliques in selection and influence processes, research in this regard is limited to the level of the entire friendship network (Sentse et al. 2013; Sijtsema et al. 2013). Therefore, we examined selection and influence processes related to bully victimization within both the larger friendship network and at the level of the clique.

### Selection and Influence Regarding Bully Victimization

Although victims of bullying are usually low in peer acceptance (de Bruyn et al. 2010; Scholte et al. 2009), there may be reasons why adolescents could select friends who are bully victimized. First, adolescents who are bully victimized themselves presumably have the need to form intimate relationships like anyone else (Baumeister and Leary 1995). Befriending others who are bully victimized might be their only option, and might be considered a default friendship choice (cf. Scholte et al. 2009; Sijtsema et al. 2013). That is, because adolescents who are bully victimized hold a relatively marginal position at school, the pool of possible peers that they can establish friendships with consists largely of peers with the same social position. Second, adolescents who are bully victimized might select friends that are also bully victimized by deliberate choice. Selecting friends who are bully victimized may be uniting (Salmivalli et al. 1997) as victims may feel understood and supported by a friend who has similar experiences. In addition, when a group perceives they are bully victimized, this may increase trust within that group, hence victims of bullying may feel connected to others who are also bully victimized, and joining their clique may be beneficial (Rotella et al. 2013). Another benefit of befriending others who are bully victimized may be that adolescents who are bully victimized are more willing to intervene against bullying, and adolescents who are bully victimized by the same perpetrators tend to defend each other (Batanova et al. 2014; Huitsing et al. 2014). Overall, we thus hypothesize that adolescents select their friends based on their level of bully victimization. We expect selection to occur both at the level of the larger friendship network and at the level of the clique.

On the one hand, selecting friends who are bully victimized may thus be beneficial. On the other hand, it can pose a risk as well. Because bully victimized adolescents tend to have poor social and emotion regulation skills as well as higher levels of psychopathological symptoms (both internalizing and externalizing), friendships between adolescents who are bully victimized might not be as beneficial as other friendships (see Prinstein and Giletta, in press). In this regard, research has shown that friendships of bully victimized adolescents have lower positive qualities and involve higher levels of conflict (Bagwell and Schmidt 2011), which eventually might lead these relationships to be short-lived (see Sijtsema et al. 2013). More importantly, these friendships might further maladaptive cognitions (e.g., self-blame, negative attribution styles) and symptomatology associated with bully victimization (Prinstein and Giletta, in press), and ultimately exacerbate the likelihood of experiencing bully victimization (via influence processes) as well.

Influence processes also may lead to similarities between friends in general, or clique members specifically, in their level of bully victimization. Friends who are bully victimized may not be adequate protectors against bullying, but rather serve as a risk factor for future bullying. One reason why friends who are bully victimized may be a risk factor for future bully victimization, is that having friends who are bully victimized may reduce opportunities to learn adequate social behavior and increase maladaptive behavior. Being bully victimized is related to low social skills (Schwartz et al. 1993). Friends who have low social skills may not be able to serve as role models of competent social behavior that might protect adolescents from bully victimization (Scholte et al. 2009). Instead, maladaptive behavior could be reinforced, creating a negative circle of maladaptive behavior (cf. cumulative continuity; Caspi et al. 1989).

Another reason why friends who are bully victimized could pose a risk for future bully victimization is that social contagion of bully victimization might occur. Joining a group with a certain social status may result in obtaining that social status as well (Peters et al. 2010; Witvliet et al. 2010a). This idea of social contagion may also apply to bully victimization. A group of adolescents who are bully victimized may hold a low social status, and have the reputation of not being able to defend themselves adequately. This may lead to acquiring a similar social position, and ultimately to an increased risk of bully victimization. Studies indeed suggest that having friends who are unable to protect against bullying, or receiving peer nominations from others who are bully victimized are risk factors for bully victimization (Hodges et al. 1997; Pellegrini et al. 1999). In addition, longitudinal friendship network analyses suggests that having a friends who is bully victimized and defending victims increase the likelihood of becoming bully victimized (Faris and Felmlee 2014; Huitsing et al. 2014). Thus, we expect that influence in terms of bully victimization would occur both at the level of the larger friendship network, and at the level of the clique.

### Gender Differences

A large body of research suggests that bullying processes might be different for boys and girls (e.g., Bjorkqvist et al. 1992; Veenstra et al. 2005). Selection and influence of bully victimization in cliques may be different for boys and girls as well. Female victims of bullying usually have a broader friendship network than male victims of bullying, in the sense that their network does not just consist of victims or otherwise rejected adolescents (Salmivalli et al. 1997). Also, girls typically show more willingness to intervene against bullying than boys, and take up the role of defender of victims more often (Batanova et al. 2014; Salmivalli et al. 1996). In addition, boys are more similar than girls in their level of bully victimization (Hodges et al. 1997). This indicates that girls may not take victim status into account in forming friendships as much as boys do. Girls also may have the opportunity to practice a larger range of social skills as their cliques are more diverse and include peers who might be able to defend against bullying. Therefore, we hypothesize that both selection and influence of friends based on the level of bully victimization will be stronger for boys than for girls.

## Present Study

The goal of this study was to examine selection and peer influence processes in relation to bully victimization. With a two-wave design, we examined selection and influence in the larger network including all friendships using stochastic actor-based modeling (Snijders et al. 2010). This analytic approach offers the unique opportunity to investigate how adolescents’ friendship network and their levels of bully victimization co-develop over time, thus allowing to simultaneously estimate selection and influence effects in friendship networks that include multiple overlapping relationships. Notably, these models also allow to control for structural network effects, such as the tendency of adolescents to become friends with the friends of their friends (i.e., transitivity effect; see Method section), which if neglected, may lead to overestimating selection as well as influence effects (Snijders et al. 2010; Veenstra et al. 2013). Earlier research using this approach found evidence for both selection and peer influence processes involved in bully victimization at the level of the entire friendship network (Sijtsema et al. 2013). Therefore, we expected to replicate this finding. Subsequently, within the broader friendship network, we identified smaller cliques. We examined whether selection and peer influence of bully victimization also occurred at the level of these cliques. We expected adolescents to select clique members based their level of bully victimization, and that clique members would influence each other’s level of bully victimization over time. Moreover, we expected that selection and influence effects would be stronger for boys than for girls.

## Methods

### Participants and Procedure

The sample consisted of 543 adolescents enrolled in four secondary schools in The Netherlands. In total, 664 students were registered at one of the four schools for secondary education included in this study. Of these students, 606 (91.3 %) completed the questionnaire. Informed consent was obtained from all individual participants included in the study. One student declined to fill out the questionnaire at T1, the other missing data were due to illness. Of the 606 participants at T1, 543 (89.6 %) also completed data at T2, 5 months later. Two students declined to fill out the second questionnaire and five students moved to another school, the other missing data were due to illness. *t* tests showed that participants who were present at both time points did not differ in terms of gender, ethnicity, education level or level of victimization from participants who dropped out.

Of the final sample, 272 were male (50.1 %). The majority of the sample had a Dutch ethnic background (92.3 %). At T1, participants ranged in age from 14 to 18 years (*M* = 15.8, *SD* = .70). The Dutch secondary school system distinguishes between education levels. In our sample, 37.2 % of the students had a low education (vocational) level, 25 % had a middle education level and 37.8 % had a high education (preparatory university) level.

Schools were informed about the research through written and personal communication. Passive parental consent was obtained for all students registered at these schools. Informed consent was obtained from all individual participants included in the study. Identical survey data were collected in fall and spring of the fourth year of secondary education. Movie vouchers were raffled among students who participated. Questionnaires were filled out during regular school hours (50 min).

### Measures

#### Bully Victimization

Before students answered questions about bully victimization, we provided them with a definition: “Bullying is when a student or a group of students says unpleasant or mean things to another student. It is also bullying when a student is being hit, beaten, threatened or locked up or other hurtful things like that. It is bullying when those things happen regularly and it is difficult for the student being bullied to defend him or herself. It is NOT bullying when two students who are equally strong quarrel, fight or tease each other.” This definition is commonly used (cf., Solberg and Olweus 2003). Bully victimization was assessed using an adapted version of a Dutch translation of the victim scale of the Olweus Bully–Victim questionnaire (Olweus 1989). The scale consisted of three items (i.e.: “How often did other students bully you in the past few months?”, “How often do other students say mean things to you?”, “How often were you hit, kicked, locked indoors, or other hurtful things like that?”). Responses were given on a 5 point scale (1 = “never”, 2 = “sometimes” 3 = “often times” 4 = “once per week” 5 = “several times per week”). The reliability was .61 at T1 and .82 at T2.

Because stochastic actor-based models require the behavioral outcomes to be ordinal variables, we used the mean of the three victimization items to create five groups, (scores per group were 1–1.32; 1.33–1.66; 1.67–1.99; 2.00–2.5; >2.5). This means that the group with the lowest levels of bully victimization indicated that they were never victimized, and the group with the highest levels of bully victimization indicated that they were victimized sometimes to oftentimes.

To obtain clique scores for bully victimization, for each participant we averaged the reported levels of bully victimization of his or her clique members excluding the participant’s own scores. This procedure is comparable to other studies to construct higher order or group scores (Sentse et al. 2007).

#### Friendships

In the Dutch school system, adolescents are part of a root class with whom they spend most of their time and follow most of their classes. Participants were given a roster with the names of all peers in their grade, sorted by root class, and preceded by an identification number. Participants were asked to nominate the peers they considered their closest friends by writing down their identification numbers. Nominations were limited to a maximum of 20. These nominations were used to create friendship networks within each grade at each time point. To do so, an adjacency matrix was created for each grade, containing information on friendship nominations and non-nominations of all possible dyads within the entire grade. Specifically, each matrix consisted of n rows by n columns (n = grade size), representing adolescents who gave nominations (i.e., nominators) and those who received nominations (i.e., nominees) respectively. The presence of a directed friendship tie from a nominator to a nominee (e.g., participant A nominated participant B as friend) was indicated by a one and the absence of such a tie by a zero (e.g., A did not nominate B). Adjacency matrices were employed in stochastic actor-based models. Friendship nominations also were used to identify cliques (see below).

#### Cliques

Cliques were established through friendship nominations. Whereas in stochastic actor-based models selection and influence processes were analyzed in the entire friendship network, based on all friendship nominations within a grade, in the clique analyses selection and influence processes were estimated within tight groups of friends (i.e., cliques) that were embedded in the larger network. To determine which cliques existed within the larger friendship network, we used the 2 2-clique procedure in UCINET 6.0 (Borgatti et al. 2002). This program produces groups of adolescents who are tightly connected through mutual nominations. Comparable to the stochastic actor-based models, adolescents are oftentimes connected to multiple groups of friends that partly overlap. To limit statistical dependency in the data, we used several decision rules to ascribe unique group membership to all participants: (1) each clique consisted of at least three friends (i.e., dyads were excluded); (2) all members of a clique had to be connected through either a direct link (i.e. clique member 1 nominated clique member 2, and clique member 2 nominated clique member 1) or an indirect link (i.e. person 1 nominated person 2, and person 2 nominated person 3); (3) two clique members could not be separated by more than 1 indirect link; (4) if a person was part of more than one clique, the clique in which she or he had the most ties was chosen. These rules are comparable to those used in earlier research (e.g., Espelage et al. 2003).

To differentiate stable and unstable cliques, we defined cliques as stable when 80 % of the T1 clique members were still in the clique at T2. This estimate of stability is more conservative than the estimate used by Ennett and Bauman (1994) who first differentiated stable and unstable cliques in order to disentangle selection and influence effects. Ennett and Bauman (1994) considered cliques stable when at least 50 % of the clique was still present at T2. However, this implies that if in a small clique of four people, two members change cliques, this clique would still be considered as stable. With our criterion of 80 %, in small cliques of three or four adolescents, all adolescents would still have to be in the same clique at T2 in order to be considered a stable clique. With five clique members at T1, only 1 clique member can have changed cliques at T2 in order to be called a stable clique. Only when the clique has at least 10 members, two clique members can change cliques between T1 and T2 for the clique to be considered stable. Thus, unstable cliques are cliques that are present at T1, but dissolve at T2 (dissolved cliques), and cliques that were not yet present at T1, but were newly formed at T2 (newly formed cliques).

### Analytic Strategy

Stochastic actor-based models were estimated using the RSiena package (Ripley et al. 2012). Specifically, the co-evolution of adolescent friendship networks and their report of bully victimization was examined over the two discrete time points using a continuous-time Markov Chain Monte Carlo (MCMC) approach. This iterative simulation procedure generated unstandardized parameters and their standard errors, from which a *t*-value is calculated. Stochastic actor-based models were estimated simultaneously across all four schools by combining them into one matrix in which structural zeros indicated that students from different schools could not nominate each other (cf. Ripley et al. 2012). More detailed information about stochastic actor-based models are available elsewhere (Snijders et al. 2010; Veenstra et al. 2013).

First, we examined the descriptive statistics of the networks and bullying data to ensure that they were suitable for friendship network analyses (see Table 1). We then estimated a model with the friendship network and being bullied as dependent variables. Two sets of parameters were estimated, one for the prediction of changes in friendship ties (i.e., network dynamic effects) and one for the prediction of changes in adolescent bully victimization (i.e., bully victimization dynamic effects). The first set of parameters included bully victimization effects (i.e., ego, alter and selection similarity effects), in order to investigate whether adolescents’ levels of bully victimization affected friendship dynamics, and in particular whether adolescents tended to select as friends peers with similar levels of bully victimization (i.e., selection similarity effect). Moreover, we controlled for basic structural network effects, including reciprocity (i.e., the tendency to reciprocate a friendship tie), transitivity triplets (i.e., the tendency to befriend friends of friends), 3-cycles (i.e., the tendency toward generalized reciprocity) and geodesic distance-two effects (i.e., the tendency to avoid befriending friends of friends), as well as other actor attribute effects related to adolescent sex, age, ethnicity and classroom. The second set of parameters included the peer influence effect on bully victimization (i.e., average similarity). Moreover, we controlled for basic tendency effects (i.e., linear and quadratic shape) as well as the main effects of sex, age and ethnicity on bully victimization dynamics (for a detailed description of these effects, see Veenstra et al. 2013).

**Table 1:** Descriptive of friendship network and bully victimization across time

	Time1	Time2
Friendship		
Number of ties	3432	3426
Average outdegree	6.32	6.31
Density	0.012	0.012
Reciprocity	59.7 %	58.9 %
Transitivity	33.3 %	32.2 %
Bully victimization		
1–1.32^a^	46.4 % (n = 252)	51.6 % (n = 279)
1.33–1.66^a^	34.3 % (n = 186)	29.4 % (n = 159)
1.67–1.99^a^	12.3 % (n = 67)	8.9 % (n = 48)
2–2.5^a^	4.6 % (n = 25)	4.4 % (n = 24)
>2.5^a^	2.4 % (n = 13)	5.7 % (n = 31)
Moran’s index	0.07	0.14

	Time1–Time2
Friendship change	
Distance	2492
Jaccard index	0.47
Changes in bully victmization	
Stable actors	54 % (n = 293)
Decreasing actors	24.9 % (n = 135)
Increasing actors	20.8 % (n = 113)

^a^Refers to mean bully victimization score

**Fig. 1:**
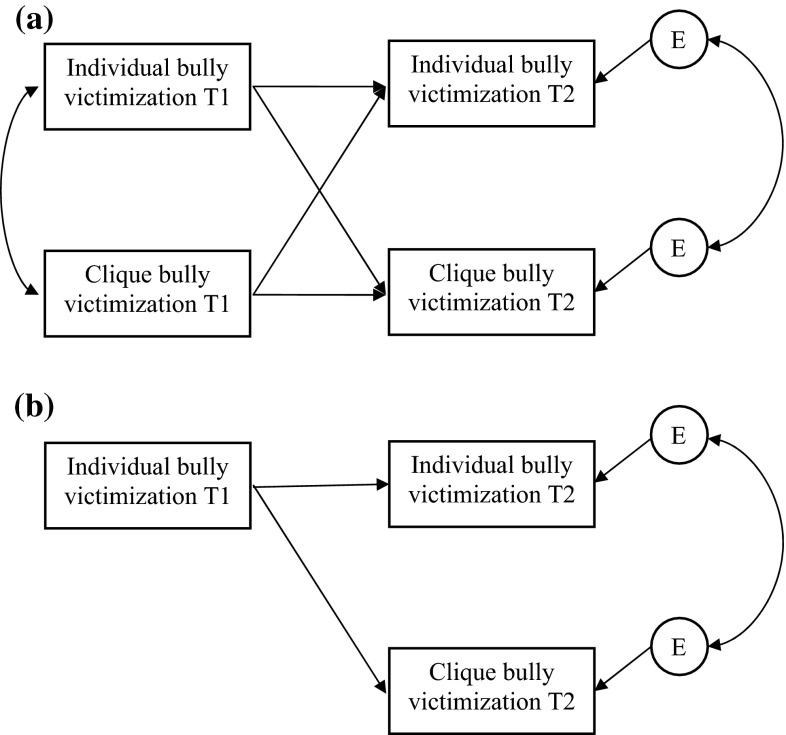
APIM models to test selection and influence effects. **a** The model to test influence effects, **b** the model to test selection effects

Second, we focused on the clique level. We estimated peer influence effects with the Actor–Partner Interdependence Model (Cook and Kenny 2005). This model (Fig. 1a) was tested for stable cliques, because differences in T2 individual reports of bully victimization that can be attributed to differences in T1 clique reports of bully victimization are likely to be due to influence rather than selection when group members did not change (Popp et al. 2008). In order to control for similarity between adolescents and their clique members, we let individual reports of bully victimization and average clique reports of bully victimization be correlated at T1 and T2. Because we hypothesized that the effect of T1 average clique reports of bully victimization on T2 individual reports of bully victimization would be different for boys and girls, we ran multiple group analysis. Subsequently we tested the same cross-lagged panel model for dissolved cliques, where we expected no significant effects of individuals on cliques or vice versa.

To test whether selection based on bully victimization occurred, we tested whether T1 individual bully victimization predicted T2 average clique bully victimization, for newly formed cliques. The model is shown in Fig. 1b. Again, we tested for moderation by gender using multiple group analyses.

## Results

### Descriptive Statistics

Table 1 presents descriptive statistics of friendship network and adolescent bully victimization. There were sufficient changes in friendships and bully victimization over time to estimate selection and influence effects. All other network and behavioral characteristics emerged adequate to carry out the analyses (Veenstra et al. 2013). Table 2 describes the means and standard deviations for all variables at T1 and T2, separately for boys and girls. Individual and clique bully victimization did not differ by gender at T1, but were slightly higher for boys than for girls at T2. In addition, we examined whether clustering of bully victimization occurred at the school level, using the Intraclass Correlation (ICC). ICC was .016 (1.6 %) at T1 and .032 (3.2 %) at T2. Thus, at both time points, clustering of bully victimization at the school level was well below the 5 % threshold, which means that it is not necessary to take it into account in further analyses (Peugh 2010; Satorra and Muthen 1995).

**Table 2:** Means and standard deviations for all measures of bully victimization

	T1			T2		
	Male	Female	T	Male	Female	T
	M (SD)	M (SD)		M (SD)	M (SD)	
Self-report	1.32 (.51)	1.30 (.41)	.62	1.46 (.84)	1.28 (.51)	3.12**
Clique	1.30 (.26)	1.27 (.20)	1.51	1.37 (.42)	1.27 (.28)	2.85**

N = 272 for males. N = 271 for females

** *p* < .01; *** *p* < .001

### Stochastic Actor-Based Model Analyses

Table 3 shows the results of the stochastic actor-based model. The general network dynamics were as expected (see Veenstra et al. (2013) for a detailed review). Ego effects (effects of individual attributes on number of nominations *given*) indicated that the number of friends who were nominated was related to age and to the level of bully victimization. Older adolescents, and adolescents reporting higher bully victimization, tended to nominate more friends. Alter effects (effects of individual attributes on number of nominations *received*) indicated that sex, age, and bully victimization did not influence the number of nominations received from peers (e.g., adolescents reporting high bully victimization received as many friendship nominations as adolescents reporting low bully victimization).

**Table 3:** Parameter estimates for stochastic actor-based model

Parameters	Estimate	S.E.
*Network dynamics*		
Structural network effects		
Reciprocity	2.05***	0.06
Transitivity triplets	0.28***	0.02
3-cycles	−0.32***	0.03
Geodesic distance-2	−0.20***	0.01
Ego effects		
Sex	−0.01	0.05
Age	0.14***	0.03
Bully victimization	0.14***	0.04
Alter effects		
Sex	0.07	0.04
Age	−0.00	0.03
Being bullied	0.05	0.03
Selection effects		
Sex similarity	0.46***	0.04
Age similarity	0.04	0.16
Same class	0.74***	0.04
Same ethnicity	−0.02	0.06
Being bullied similarity	0.43*	0.20
*Bully victimization dynamics*		
Linear shape	−0.59***	0.08
Quadratic shape	0.29***	0.05
Average similarity (influence)	2.29*	0.93
Effect from sex	−0.26**	0.10
Effect from age	−0.04	0.08
Effect from ethnicity	−0.13	0.19

* *p* < .05; ** *p* < .01; *** *p* < .001

Selection effects indicated that participants tended to select friends who were of the same gender, in the same classroom, and had similar levels of bully victimization. Thus, in the larger friendship network there was indeed a selection effect of bully victimization. The bully victimization dynamics showed that most participants scored below the mean on bully victimization (negative linear shape). Moreover, adolescents who reported higher bully victimization at T1 tended to increase in bully victimization even more over time, as compared to adolescents who reported lower bully victimization at T1 (positive quadratic shape). In addition, the average similarity parameter indicated that adolescents tended to become more similar to their friends in terms of bully victimization over time, thereby providing evidence for influence processes. Finally, the negative effect of the sex parameter indicated that boys increased more in bully victimization over time compared to girls. Together, the network analyses indicated that adolescents tended to select friends who were similar to them in terms of bully victimization (selection), and once these friendships were formed, adolescent friends tended to become more alike in bully victimization (influence).

### Clique Membership

Next, we examined selection and influence effects of bully victimization at the clique level. At T1, 449 participants (82.7 %) were part of a clique; 448 participants (82.5 %) were part of a clique at T2. Cliques ranged in size from 3 to13 (*M* = 4.92, *SD* = 2.40) at T1 and from 3 to 11 (*M* = 4.94, *SD* = 2.58) at T2, which is comparable to earlier studies (e.g., Ennett and Bauman 1994). We found that 204 participants (37.6 %) were part of a stable clique.

### Influence at the Clique Level

We used the Actor–Partner Interdependence Model depicted in Fig. 1a to test whether clique members influenced each other’s levels of bully victimization. Multiple group analyses showed that the model with all parameters constrained to be the same for boys and girls was significantly different from the model with all parameters estimated separately for boys and girls ($\upchi_{\text{diff}}^{2} = \, 42.402$, *df*_diff_ = 6, *p* < .001). To examine what paths were significantly different for boys and girls, we tested each restricted path against the model with only free paths. We found that there was a difference in stability of individual reports of bully victimization ($\upchi_{\text{diff}}^{2} = \, 4.30$, *df*_diff_ = 1, *p* = .038), stability of the average clique report of bully victimization ($\upchi_{\text{diff}}^{2} = \, 22.10$, *df*_diff_ = 1, *p* < .001), the error correlation at T2 ($\upchi_{\text{diff}}^{2} = { 7}.90$, *df*_diff_ = 1, *p* = .005) and the association between T1 average clique reports of bully victimization and T2 individual reports of bully victimization ($\upchi_{\text{diff}}^{2} = \, 22.10$, *df*_diff_ = 6, *p* < .001). The final model shown in Table 4 had good fit (χ^2^(2) = 2.83, *p* = .243, CFI = 1.00, RMSEA = .05). The results imply that for boys who are a member of a clique with high average levels of bully victimization, the likelihood of those boys to become a victim of bullying at T2 are higher. Being a part of a clique whose members report low average levels of bully victimization at T1 decreases the level of bully victimization at T2. For girls, these effects were not significant. Thus, there was influence of the clique on the individual for boys but not for girls.

**Table 4:** Standardized estimates and standard deviations for APIM models

Predictor	Girls		Boys	
	β	SE	β	SE
Cross-lagged paths				
Individual T1 → Clique T2	.09	.05	.09	.05
Clique T1 → Individual T2	−.21	.23	.46**	.15
Cross-sectional association				
Individual T1 ↔ Clique T1	.02***	.01	.02***	.01
Individual T2 ↔ Clique T2	.05***	.01	−.00	.01
Stability paths				
Individual T1 → Individual T2	.58***	.14	.87***	.09
Clique T1 → Clique T2	.31	.13	1.16***	.09

** *p* < .01; *** *p* < .001

Regarding the influence of individuals on cliques, for both boys and girls individual reports of bully victimization at T1 did not predict average clique members’ reports of bully victimization T2. Thus, when clique members on average report high levels of bully victimization, having one group member with low levels of bully victimization does not decrease the risk of bully victimization. In addition, having one clique member who reports high levels of bully victimization does not increase bully victimization for the other clique. Additionally, the stability of individual and average clique members’ reports of bully victimization was higher for boys than for girls. Being the victim of bullying at T1 increased the chance of being the victim of bullying at T2 more for boys than for girls.

Subsequently, we tested the same models for dissolved cliques. We hypothesized that average clique members’ reports of bully victimization at T1 would not influence individual reports of bully victimization at T2. The model did not differ between boys and girls ($\upchi_{\text{diff}}^{2} = { 6}.94$, *df*_diff_ = 6, *p* = .326). Also, the cross-lagged paths indicating influence did not reach significance. Thus, in cliques that were dissolved at T2, there was no influence of clique members on individuals or of individuals on clique members in terms of bully victimization.

### Selection at the Clique Level

The model in Fig. 1b was run to test for selection effects at the clique level. The model did not include a path from T1 average clique reports of bully victimization to T2 average clique reports of bully victimization, because the clique does not exist yet at T1. The models for selection were tested for newly formed cliques only because selection can only be assessed in cliques that are established between T1 and T2. Multiple group analyses showed no gender differences ($\upchi_{\text{diff}}^{2} = { 5}.61$, *df*_diff_ = 3, *p* = .132). For the model in Fig. 1b, the path from T1 individual reports of bully victimization to T2 average clique members’ reports of bully victimization did not reach significance. Adolescents did not select their clique members based on their level of bully victimization.

## Discussion

Earlier research emphasized the importance of group processes in bully victimization (Salmivalli 2010). Adolescents reporting bully victimization may actively select friends who are also bullied, because they are the default choice (cf. Scholte et al. 2009; Sijtsema et al. 2013), or by deliberate choice (Huitsing et al. 2014; Salmivalli et al. 1997). In addition, adolescents may influence their friends’ levels of bully victimization over time, because friends who are bullied may not provide opportunities to practice social skills needed to defend against bullying (Scholte et al. 2009), or because of social contagion of bully victimization status (Faris and Felmlee 2014; Huitsing et al. 2014). Indeed, earlier research showed that selection and influence processes play a role in bully victimization at the level of the larger friendship network (Sentse et al. 2013; Sijtsema et al. 2013). Earlier research also indicated that different types of friendships exist within the larger friendship network, and that, in some instances, closer friends may be of larger influence than more distant relations (Giletta et al. 2012). Cliques may be especially important, because they encompass close friendships, and clique members are amongst the most important peers for adolescents (Bagwell et al. 2000; Conway et al. 2011; Espelage et al. 2007; Thompson et al. 2001).

Our study was the first to examine selection and influence processes involved in bully victimization at the level of the larger friendship network, and at the level of friendship cliques. We hypothesized that selection and influence of bully victimization would occur both levels, and that selection and influence of bully victimization at the clique level would be stronger for boys than for girls. Indeed, in the larger friendship network there was evidence that adolescents select friends based on their level of bully victimization, and influence the degree to which their friends’ levels of bully victimization over time. Contrary to expectations, at the clique level adolescents did not select their clique members on the basis of these members’ levels bully victimization. For boys, we found evidence suggesting that the average level of victimization in a clique influences the level of individual bully victimization over time. This influence effect was not found for girls at the level of the clique. In addition, whereas we found that average clique levels of bully victimization influenced future individual levels of bully victimization, we did not find that adolescents’ individual levels of bully victimization influenced average clique levels of bully victimization.

Our findings for the larger friendship network replicated earlier findings indicating that both selection and influence processes account for similarities between friends’ levels of bully victimization (Sentse et al. 2013; Sijtsema et al. 2013). Thus, in general adolescents tend to befriend others with similar levels of bully victimization, and they tend to become more alike in bully victimization over time. Regarding cliques, cross-sectional studies suggest that both selection and influence processes are responsible for similarities between clique members’ level of bully (Salmivalli et al. 1997). Our findings, using two time points, indicate that similarity between clique members’ bully victimization may not be due to selection. Thus, although selection was observed within the larger network that included all friendship ties, including for instance less close relations with friends who were not in the same clique, such selection effects did not hold for cliques. As cliques consist of relatively close friendships with whom adolescents spend most of their time (Brown 2004; Brown and Klute 2006; Henrich et al. 2000), selection of friends who are bully victimization to the same extent may thus not be due to the selection of one’s closest friends, but rather seem to reflect a tendency to select friends from a larger pool of friends that hold a similar social status. Thus, whereas adolescents’ selection of any friend within their grade may be influenced by their bully victimization levels, this does not necessarily apply to the selection of their closest friends. This is an important finding as it counteracts the idea that adolescents who are bullied may actively and deliberately select very specific social niches that pose risks for prolonged bullying. Future research could explore this idea by examining selection and influence effects at the level of the best friend, and by combining research on selection and influence processes involving bullying and social status.

We found evidence for peer influence regarding bully victimization in the larger friendship network, in line with earlier findings (Sentse et al. 2013; Sijtsema et al. 2013). At the clique level, our findings indicated peer influence regarding bully victimization only for boys. For boys, the average level of clique members’ bully victimization predicted predicted individual levels of bully victimization over time. Thus, for boys social contamination processes seem to occur that increase the likelihood of bully victimization by associating with other victims. A reason for this may be that friends of adolescents high in bully victimization acquire a similar social position and are seen as easy targets who are not likely to retaliate successfully against harassment (Hodges et al. 1997; Witvliet et al. 2010a, b). In addition, peers with high levels of bully victimization may be inadequate role models who cannot help to acquire the social skills needed to defend against bully victimization and may instead reinforce socially maladaptive behavior (Scholte et al. 2009).

One crucial point to be addressed in future research is the question when peer groups become a risk for bully victimization and when they serve a protective function. Our findings indicate that if a clique consisting of adolescents who are not bullied are joined by one adolescent high in bully victimization, this does not seem to increase the likelihood that the other clique members will become the victim of bullying as well. At the same time, if a adolescent who reports low levels of bully victimization is part of a clique with high levels of bully victimization this does not provide protection for the entire clique. This finding further stresses the importance of incorporating groups in our understanding of adolescent bully victimization. Group factors (i.e., whether adolescents’ clique members are bully victimized of not) are of great importance for the future bully victimization status of individuals, whereas individuals do not influence the clique as much.

We only found evidence for influence of clique members in stable cliques, that is, in cliques that were still present in the same composition at T2, and not in cliques that were dissolved at T2. This is in line with findings on dissolved friendships in earlier research (Laursen et al. 2012). A reason why cliques break up might be the extent to which some of their members are bully victimized, as bully victimization is related to de-selection of friends (Sijtsema et al. 2013). Adolescents may be aware of the risk of being part of a clique characterized by high levels of bully victimization, and decide to diminish this risk is by leaving the clique. In addition, clique members may exclude specific others in their clique who they perceive are high in bully victimization. As Bukowski and Sippola (2001) suggested, peer groups have goals such as group cohesion and homogeneity. Adolescents high in bully victimization may jeopardize these group goals, for example by threatening cohesion because other members experience increased risk of bully victimization. Excluding this clique member may thus be beneficial for the clique. Although such processes have been proposed for aggression and the perpetrators of bullying (Garandeau and Cillessen 2006), development of cliques in relation to bully victimization needs to be addressed in future research.

Regarding gender differences, we confirmed our hypothesis that influence effects are stronger for boys than for girls. In fact, we found no evidence for peer influence processes relating bully victimization in adolescent cliques for girls at all. This might be due to the differences in networks between boys and girls. Girls have more diverse networks in terms of bully victimization than boys; for instance, it is possible for girls to have both perpretrators and victims of bullying in their network (Salmivalli et al. 1997). This indicates that the proposed processes might not be as apparent for girls as for boys. Girls may have different role models in their clique (i.e., not just adolescents with low social status or low social skills), so the negative cycle of maladaptive behavior might not occur. Moreover, girls’ clique members might be more able to defend each other than boys’ clique members. Indeed, research suggests that girls are more likely to take up the role of defender in bullying situations than boys (Batanova et al. 2014; Salmivalli et al. 1996).

This gender difference also has implications for prevention and intervention programs against bullying. In recent programs, peers have been used to prevent or intervene against bullying, for instance by providing support groups for victims of bullying (Ttofi and Farrington 2009). For boys, clique members may only be effective against bully victimization if these clique members are low in bully victimization themselves. If boys have clique members who are all high in bully victimization, selecting other peers from the larger friendship network in a support group may be a better strategy. For girls, clique members do not seem to influence the degree to which adolescents are bullied. For girls, having clique members who are high in bully victimization is not a risk factor, but having clique members who are low in bully victimization is not a protective factor either. Future research could examine whether it is beneficial for girls to train clique members to intervene against bullying, or whether only peers from the larger friendship network, such as popular classmates, are effective in reducing bullying (Faris and Felmlee 2014). Moreover, because we found evidence for selection and influence at the level of the larger friendship network, our results imply that adolescents may select friends from a larger pool of friends that hold similar social positions, and that they may be influenced by others in the same general social group as well. This emphasizes the importance of social position for interventions against bullying.

A major strength of the present study is that we examined selection and influence regarding bully victimization at the level of the entire friendship network and at the level of cliques. This allowed us to obtain a more in depth view of where selection and influence processes involved in bully victimization for adolescents. Moreover, our study is the first to examine selection and influence processes for bully victimization in the context of cliques using multiple time points.

Despite its strengths, this study also had some limitations. First, we did not include peer reported bully victimization in our design. One of the mechanisms we proposed behind clique members’ influence on individual levels of bully victimization is social contamination, which means that adolescents might become perceived by peers as victims of bullying when their clique members are bullied. Future research should include peer reported bully victimization to explore this option. Second, we did not differentiate between various forms of bully victimization, whereas earlier research suggested that selection and influence processes may differ for overt and relational bully victimization (Sijtsema et al. 2013). Future research should examine whether such differences hold for selection and influence processes in cliques as well. Third, we only included two time points that were 5 months apart. Although this is a relatively short interval, we cannot establish what changes in the network may have occurred between the two time points. For instance, cliques that appear to be stable may have been broken up for a while. Although the method we used to examine peer influence is common (Popp et al. 2008), we cannot be certain that similarities between clique members’ levels of bully victimization are actually due to influence processes, rather than selection processes, for boys. Only stochastic actor-based modeling allowed to clearly disentangle selection and influence effects, because in this approach unobserved changes between discrete observations were simulated (see Steglich et al. 2010) (see Steglich et al. 2010). Future research should thus include more time points and shorter intervals.

## Conclusion

Our results confirmed that, at the level of the larger friendship network, adolescents tend to select friends based on their level of bully victimization, and that friends influence each other’s levels of bully victimization over time. However, at the level of the clique, we found no evidence for selection based on bully victimization, and we only found evidence for influence on bully victimization for boys. This implies that adolescents may form friendships within a larger pool of peers holding similar social position, based on these peers’ levels of bully victimization. However, they may not intentionally select their closest friends (clique members) who are high in bully victimization, thus, becoming friends may be a default rather than a deliberate choice (Huitsing et al. 2014; Salmivalli et al. 1997; Scholte et al. 2009; Sijtsema et al. 2013). In addition, our findings imply that for boys, being in a clique with high levels of bully victimization is a risk for future individual levels of bully victimization. This could be due to obtaining a similar social (victimization) position, or restricted abilities to practice social skills with one’s closest friends (Faris and Felmlee 2014; Huitsing et al. 2014; Scholte et al. 2009). Moreover, different mechanisms may underlie influence regarding bully victimization for girls and boys. For instance, girls may be more likely to defend their clique members compared to boys (Batanova et al. 2014; Salmivalli et al. 1996). Based on our findings, future research should take into account different types of friendship ties, in which differentiating between more distant and closer friends is essential.
